# Supplementary material for: Delivery of siRNA to Ewing Sarcoma Tumor Xenografted on Mice, Using Hydrogenated Detonation Nanodiamonds: Treatment Efficacy and Tissue Distribution
Source: Nanomaterials (Basel). 2020 Mar 19;10(3):553. doi: 10.3390/nano10030553 (PMC7153391; doi:10.3390/nano10030553)
Supplement: Supplementary file 1 [file nanomaterials-10-00553-s001.pdf]

Delivery of siRNA to Ewing Sarcoma Tumor Xenografted on Mice, Using Hydrogenated Detonation Nanodiamonds: Treatment Efficacy and Tissue Distribution

Sandra Claveau [1,2], Émilie Nehlig [3], Sébastien Garcia-Argote [3], Sophie Feuillastre [3], Grégory Pieters [3], Hugues A. Girard [4], Jean-Charles Arnault [4], François Treussart [1,5,†,*] and Jean-Rémi Bertrand [2,†]

1 LuMIn, CNRS, ENS Paris-Saclay, CentraleSupélec, Université Paris-Saclay, 91405, Orsay, France; sandra.claveau@live.fr (S.C.)
2 Vectorologie et Thérapeutiques Anticancéreuses, CNRS, Institut Gustave Roussy, Université Paris-Saclay, 94805, Villejuif, France ; jean-remi.bertrand@gustaveroussy.fr (J.-R.B.)
3 SCBM, Institut Joliot, CEA, Université Paris-Saclay, 91191, Gif-sur-Yvette, France ; e.nehlig@gmail.fr (E.N.) ; sebastien.garcia-argote@cea.fr (S.G.-A.) ; sophie.feuillastre@cea.fr (S.F.) ; gregory.pieters@cea.fr (G.P.)
4 Diamond Sensors Laboratory, Institut LIST, CEA, Université Paris-Saclay, 91191, Gif-sur-Yvette, France ; hugues.girard@cea.fr (H.A.G.) ; jean-charles.arnault@cea.fr (J.C.A.)
5 Institut d'Alembert, CNRS, ENS Paris-Saclay, Université Paris-Saclay, 91190, Gif-sur-Yvette, France
* Correspondence: francois.treussart@ens-paris-saclay.fr (F.T.)
† Co-senior authors

Supplementary Materials

Table S1. Determination of free tritium in nanodiamond suspension and in mice urine after injection. To evaluate the fraction of labile tritium the solutions were centrifugated during 3 h at acceleration 10600 *g* (50Ti rotor in XL90 Beckman ultracentrifuge). Aliquots of 100 µL were sampled before and after centrifugation and then diluted in 8 mL final volume of deionized water before radioactivity measurement by liquid scintillation. In the case of T-DND solution, we detected 5% of the initial deionized water dose present in the supernatant. We consider that this radioactivity is due to tritiated water, since it could be fully recovered on a paper filter after evaporation and re-condensation in a closed tube. The resulted centrifugated T-DND was the "purified T-DND" injected into the mice. Note that an additional centrifugation of this purified T-DND solution still reveals 2% of activity in the supernatant, which indicates that adsorbed tritium is still present at DND surface. Then, to determine if T-DND were present in urines, a similar centrifugation protocol was applied to 2 mL of urines collected 24 h after purified T-DND injection, and diluted in 8 mL final volume of deionized water. We did not detect a significant change of radioactivity in the aqueous phase after centrifugation. Furthermore, there was no solid T-DND pellet for all the 3 conditions tested (free T-DND and for the two T-DND coated with a siRNA).

	**DND suspension before centrifugation (counts per minutes, cpm)**	**Supernatant of centrifugation (cpm)**	**% of the initial dose present in the supernatant**
T-DND	1382796	63341	5
purified T -DND	1219936	24223	2
Urine T-DND	113	131	117
Urine T-DND:siAS	89	106	119
Urine T-DND:siCt	115	104	90

Table S2. Size measurement of hydrogenated and tritiated DND. Hydrodynamic diameters are inferred from scattered light intensity time autocorrelation. The raw data are in the first column (from left); the size "in number" (second column) is obtained from raw data after correction from Rayleigh and Mie scatterings, which reinforces the contribution of the smallest nanoparticles having a lower scattering efficiency. PDI (third column): polydispersity index. Zeta potential is displayed in the fourth column. *n.d.*: not determined. H-DND-24 and T-DND are the two samples used for *in vitro* and *in vivo* siRNA delivery.

Sample ref.	hydrodynamic diameter in intensity (nm)	hydrodynamic diameter in number (nm)	PDI	Zeta Potential (mV)
H-DND-22	75	31	0.17	+40
H-DND-23	72	31	0.21	+46
H-DND-24	63	56	0.18	+55
T-DND	93	48	0.23	*n.d.*
